# Dichloroacetate restores colorectal cancer chemosensitivity through the p53/miR-149-3p/PDK2-mediated glucose metabolic pathway

**DOI:** 10.1038/s41388-019-1035-8

**Published:** 2019-10-09

**Authors:** Yu Liang, Lidan Hou, Linjing Li, Lei Li, Liming Zhu, Yu Wang, Xin Huang, Yichao Hou, Danxi Zhu, Huimin Zou, Yan Gu, Xiaoling Weng, Yingying Wang, Yue Li, Tianqi Wu, Mengfei Yao, Isabelle Gross, Christian Gaiddon, Meng Luo, Jianhua Wang, Xiangjun Meng

**Affiliations:** 1 Department of Gastroenterology, Shanghai Ninth People’s Hospital, Shanghai Jiao Tong University School of Medicine, Shanghai, China; 2 Department of General Surgery, Shanghai Ninth People’s Hospital, Shanghai Jiao Tong University School of Medicine, Shanghai, China; 3 Cancer institute, Fudan University Shanghai Cancer Center, Fudan University, Shanghai, China; 4 Ningbo Aitagene Technology Co. LTD, Shanghai, China; 5 Department of Biochemistry and Molecular & Cell Biology, Shanghai Jiao Tong University School of Medicine, Shanghai, China; 6 Pathology Center, Shanghai First People’s Hospital, Shanghai Jiao Tong University School of Medicine, Shanghai, China; 7 INSERM UMR_S1113, Strasbourg, F-67200 France; 8 FMTS, Universite de Strasbourg Strasbourg, Strasbourg, F-67000 France; 9 Universite de Strasbourg, Inserm IRFAC UMR_S1113, Laboratory Stress Response and Innovative Therapy “Streinth”, Strasbourg, 67200 France; 10 CLCC Paul Strauss, Strasbourg, France

**Keywords:** Colorectal cancer, Non-coding RNAs

## Abstract

The development of chemoresistance remains a major challenge that accounts for colorectal cancer (CRC) lethality. Dichloroacetate (DCA) was originally used as a metabolic regulator in the treatment of metabolic diseases; here, DCA was assayed to identify the mechanisms underlying the chemoresistance of CRC. We found that DCA markedly enhanced chemosensitivity of CRC cells to fluorouracil (5-FU), and reduced the colony formation due to high levels of apoptosis. Using the microarray assay, we noted that miR-149-3p was involved in the chemoresistance of CRC, which was modulated by wild-type p53 after DCA treatment. In addition, PDK2 was identified as a direct target of miR-149-3p. Mechanistic analyses showed that overexpression of miR-149-3p enhanced 5-FU-induced apoptosis and reduced glucose metabolism, similar to the effects of PDK2 knockdown. In addition, overexpression of PDK2 partially reversed the inhibitory effect of miR-149-3p on glucose metabolism. Finally, both DCA treatment and miR-149-3p overexpression in 5-FU-resistant CRC cells were found to markedly sensitize the chemotherapeutic effect of 5-FU in vivo, and this effect was also validated in a small retrospective cohort of CRC patients. Taken together, we determined that the p53/miR-149-3p/PDK2 signaling pathway can potentially be targeted with DCA treatment to overcome chemoresistant CRC.

## Introduction

Colorectal cancer (CRC) is the fourth leading cause of cancer-related death in China [1] and is the second leading cause of cancer-related mortality in the United States [2], which mainly attributes to metastasis and chemotherapy failure due to drug resistance, leading to ~50,000 deaths annually [3].

Recently, while the emerging star PD1/PDL1 attracted great interest, and more biotherapeutic agents were showing encouraging results in cancer treatment, the limited efficacy rate and inevitable adverse effects restrain its use in the clinic [4, 5]. Currently, chemotherapy is still a major choice in the clinic, especially for patients unresectable late-stage and metastatic cancers [6], but the development of drug resistance remains the greatest limitation in chemotherapy [7]. Hence, exploring the mechanisms of drug resistance and delving novel combinations of classical anticancer drugs to optimize the efficacy may provide a benefit for the treatment of CRC. As fluorouracil (5-FU) is the most commonly used chemotherapeutic drug in CRC, 5-FU-resistant CRC cell lines were used in this study [8, 9].

A glucose metabolic abnormality represents one of the major aspects of the hallmarks of cancer [10]. It is known that the development of an uncontrolled cellular mass leads to poor vascularization of the tumor, causing a reduced oxygen supply. Hence, cancer cells adapt to the alterations in the microenviroment by shifting their metabolism from oxidative metabolism toward glycolytic metabolism, which is based on a glucose supply and produces lactate. This shift is called the “Warburg effect” and is commonly observed in various cancer cells as one of remarkable hallmarks [11–13]. The recent accumulation of research data led to the requirement of refining Warburg's theory [14]; for example, metabolic shift has been shown to be involved in chemoresistance [15]; hence, targeting cancer cell metabolism patterns could potentially be exploited to overcome chemoresistance [16, 17].

Notably, multiple mechanisms have been reported to control the metabolic shift in cancer cells, including microRNAs (miRNAs) [18–20]. miRNAs represent a class of small, endogenous noncoding RNAs that regulate the translation and degradation of mRNAs [21] and are involved in many more biological processes including cell proliferation, migration, apoptosis, self-renewal, initiation, cancer development, and chemoresistance [22–24].

The evidence indicates that targeting the abnormal metabolism of cancer cells has been an intense avenue of research aiming at “asphyxiating the tumor”, whose strategies are to inhibit key enzymes involved in glycolytic metabolism [15]. In this manuscript, dichloroacetate (DCA) was originally used to treat lactic acidosis and hereditary mitochondrial disease [25]. DCA inhibits the enzymatic activity of pyruvate dehydrogenase kinases (PDK1–4), which is necessary to transform pyruvate into acetyl-CoA, linking glycolytic metabolism to the citric acid cycle [26, 27]; DCA has recently been reported to have anticancer effects [28–31]. However, the mechanism underlying the effect of DCA on CRC treatment remains elusive.

The present study focused on the molecular mechanism involved in regulating glucose metabolism and chemotherapy resistance in CRC. Using DCA in CRC cells, we investigated the roles of related miRNAs and thereby disclosed a signaling pathway that accounts for 5-FU treatment resistance.

## Results

### DCA restores the chemosensitivity of 5-FU-resistant CRC cells

**Fig. 1:**
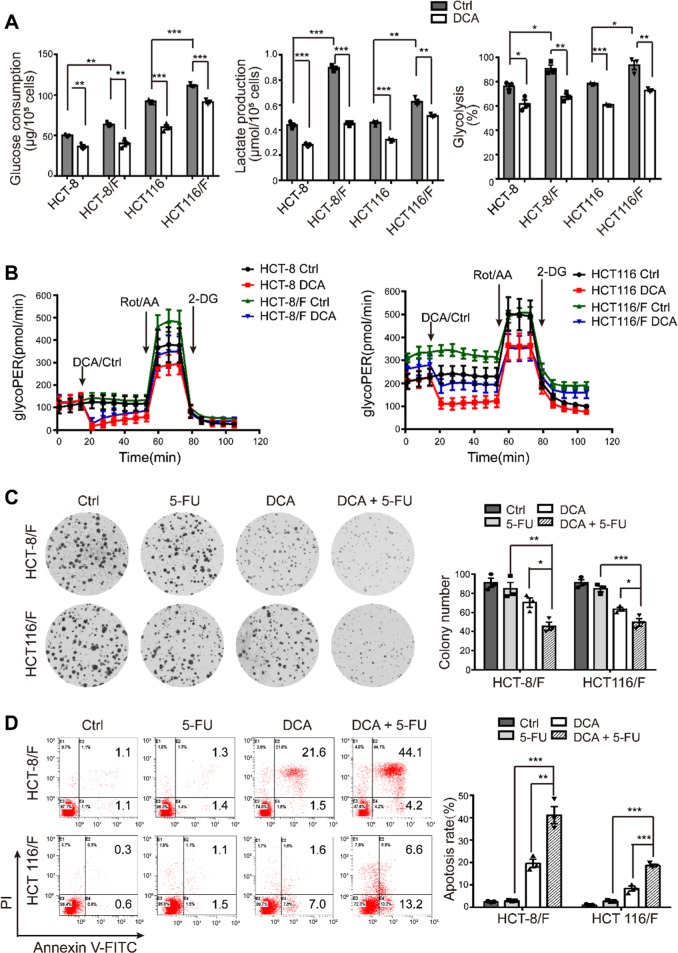
DCA restores the chemosensitivity of 5-FU-resistant CRC cells by shifting glucose metabolism. **a** HCT-8/F and HCT116/F cells were treated with 15 mM and 20 mM DCA, respectively, for 24 h. Glucose consumption, lactate production, and glycolysis of CRC cells were measured. **b** GlycoPER was measured in real time injection of 15/20 mM DCA, 0.5 μM Rot + AA, and 50 mM 2-DG using the Seahorse XF instrument. **c**, **d** HCT-8/F and HCT116/F cells were treated with DCA (15 mM)/5-FU (50 μg/ml) and DCA (20 mM)/5-FU (25 μg/ml), respectively. Colony formation assay was determined by crystal violet staining. Cell apoptosis was measured by Annexin V/PI staining. The results of three independent experiments are shown as the mean ± SEM. Each experiment was performed encompassed three biological replicates. ^*^P *<* 0.05; ^**^P *<* 0.01; ^***^P *<* 0.001

It has been reported that DCA is an effective antitumor drug that acts by targeting energy-related pathways in certain cancers [32]; however, the effect of DCA in chemoresistant CRC cells has not been well addressed. Using the CCK8 assay, we found that compared with their parental cell lines HCT-8 and HCT116 cells, 5-FU-resistant HCT-8/F and HCT116/F cells were insensitive to 5-FU (Supplementary Fig. 1A) and the half-maximal inhibitory concentrations (IC50) of DCA in HCT-8/F and HCT116/F cells were ~15 and 20 mM, respectively, which is in accord with previous reports [33, 34] (Supplementary Fig. 1B). We next noted that DCA significantly inhibited DNA synthesis (Supplementary Fig. 1C, upper panel) and induced ROS generation (Supplementary Fig. 1C, lower panel) in 5-FU-resistant CRC cells. The energy metabolism markers, including glucose consumption, lactate production, and glycolysis, were markedly elevated in 5-FU-resistant CRC cells compared with those in 5-FU-sensitive CRC cells, while addition of DCA markedly reduced the expression of those markers (Fig. 1a). Considering 6 h serum-free pretreatment in measurement of glycolysis, a Seahorse XF glycolytic rate assay kit was used to eliminate the effect of pretreatment and to measure the glycolytic rate in real time. The glycolytic proton efflux rate (glycoPER) reflects the rate of extracellular acidification form glycolysis. The addition of DCA significantly reduced the induced glycolysis compared with the respective controls (Fig. 1b and Supplementary Fig. 1D).

DCA significantly overcame 5-FU resistance in HCT-8/F and HCT116/F cells, as manifested by the measurements of cell growth (Supplementary Fig. 1E). The colony formation capacity was significantly inhibited (Fig. 1c), and the apoptosis was markedly induced in a combination treatment (Fig. 1d), all of which were further quantified. These results suggest that DCA may restore chemosensitivity in 5-FU-resistant CRC cells.

### miR-149-3p plays a crucial role in chemosensitivity in CRC cells

**Fig. 2:**
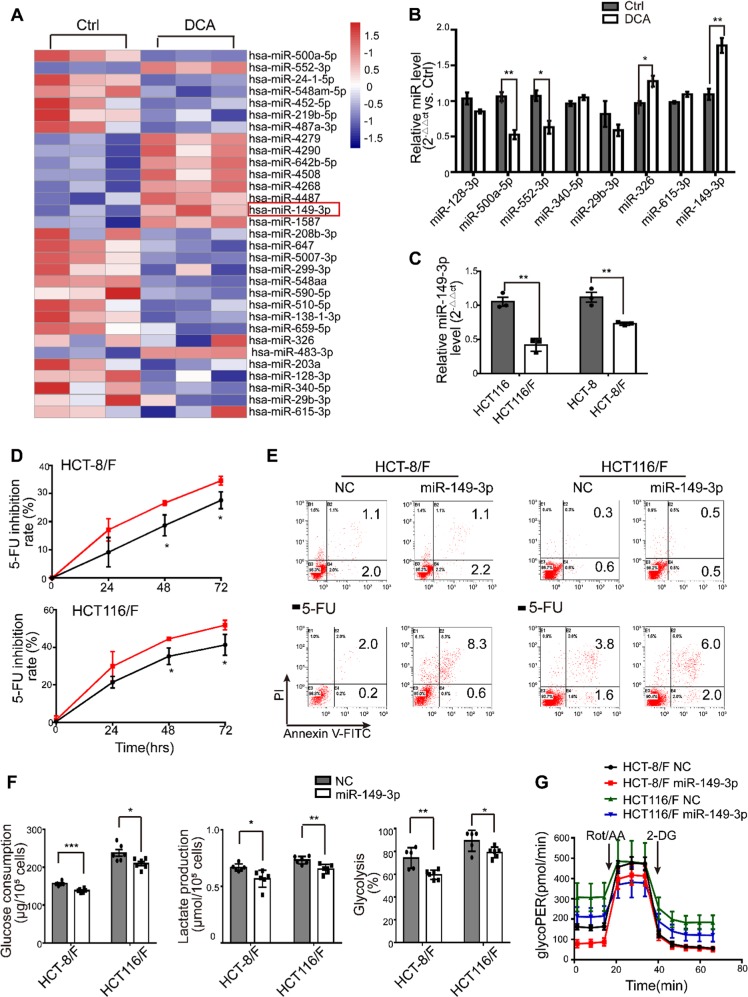
miR-149-3p enhances the chemosensitivity of 5-FU in CRC. **a** The heatmap of the differentially expressed microRNA profile in HCT116 cells treated with 20 mM DCA for 24 h. **b** Total RNA was prepared at 24 h after DCA treatment from HCT116 cells. The obviously changed microRNAs after DCA treatment were quantified by quantitative real-time PCR. **c** Total RNA was prepared from HCT116, HCT116 /F, HCT-8, and HCT-8/F cells. The basal levels of miR-149-3p were measured by quantitative real-time PCR. **d**, **e** HCT-8/F and HCT116/F cells were transiently transfected with NC and a miR-149-3p mimic for 24 h, and the cells were treated with or without 5-FU (50 μg/ml for HCT-8/F and 25 μg/ml for HCT116/F). The inhibition rate and apoptosis rate were measured. **f**, **g** The glucose consumption, lactate production, glycolysis, and glycoPER of CRC cells transfected with NC or a miR-149-3p mimic were measured. The results of three independent experiments are shown as the mean ± SEM. Each experiment contained at least three biological replicates. ^*^P *<* 0.05; ^**^P *<* 0.01

miRNAs have been considered a promising therapeutic tool for their effects on tumor suppression [35]. In this regard, we first determined miRNA expression profiles using the miRNA array containing 2059 human miRNAs. A total of 119 miRNAs were differentially expressed in response to DCA in HCT116 cells (Fig. 2a and Supplementary Fig. S2A). Among them, the expression levels of eight miRNAs were further confirmed by quantitative real-time PCR (Fig. 2b), and miR-149-3p was finally found to be upregulated by DCA in a dose-dependent manner (Supplementary Fig. S2B).

Next, we found that HCT116 cells with higher basal levels of miR-149-3p conferred more sensitivity to 5-FU and L-OHP, as shown in Supplementary Fig. 2C. Moreover, anti-miR-149-3p transfection of HCT116 cells remarkably reduced the chemotherapeutic effect of 5-FU (Supplementary Fig. 2D–E). Notably, the levels of miR-149-3p in chemosensitive CRC cells were significantly higher than the levels in chemoresistant CRC cells (Fig. 2c). Therefore, the transfection of miR-149-3p mimics significantly increased the inhibition rate; promoted cell apoptosis induced by 5-FU; reduced glucose consumption, lactate production and glycolysis in HCT-8/F and HCT116/F cells (Fig. 2d–f). A Seahorse XF glycolytic rate assay showed that miR-149-3p expression reduced the basal glycoPER in 5-FU resistant CRC cells, which were consistent with above results (Fig. 2g and Supplementary Fig. 2F). These results suggest that miR-149-3p is favorable for overcoming chemoresistance in CRC cells.

### DCA induces miR-149-3p expression through wild-type (wt) p53

**Fig. 3:**
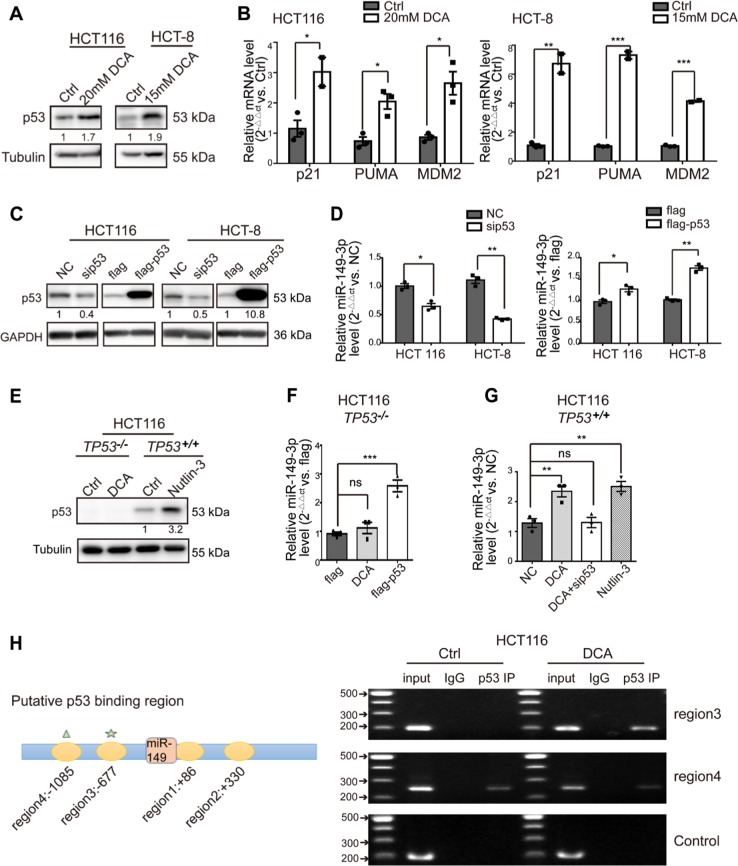
DCA induces miR-149-3p expression through p53. **a** HCT116 cells and HCT-8 cells were treated with 20 mM and 15 mM DCA, respectively, for 24 h. The expression of p53 was determined by Western blot analysis. **b** The mRNA levels of p21, puma, and MDM2 were measured by quantitative real-time PCR. **c** HCT116 cells and HCT-8 cells were transiently transfected with p53 siRNA, p53 plasmid, and the corresponding negative controls for 48 h. The expression of p53 was determined by Western blot analysis. **d** The expression of miR-149-3p was measured by quantitative real-time PCR. **e** TP53^−/−^ HCT116 cells were treated with DCA, and TP53^+/+^ HCT116 cells were treated with nutlin-3. The expression of p53 was determined by Western blot analysis. **f** TP53^−/−^ HCT116 cells were treated with DCA for 24 h or transfected with a p53 plasmid or a control plasmid. **g** TP53^+/+^ HCT116 cells were treated with DCA and nutlin-3 for 24 h or transfected with p53 siRNA after DCA treatment. The expression of miR-149-3p was measured by quantitative real-time PCR. **h** A ChIP analysis of p53 binding to the miR-149-flanking genomic DNA region from HCT116 cells treated with or without 20 mM DCA for 24 h. The results of three independent experiments are shown as the mean ± SEM. Each experiment contained three biological replicates. ^*^P *<* 0.05; ^**^P *<* 0.01; ^***^P *<* 0.001; ns no significance

Giving that several recent studies have revealed that miR-149-3p is regulated by several drugs [36, 37], we next determined how miR-149-3p is regulated by DCA. We found that DCA significantly increased the expression of wt p53 and its downstream signals, including the expression of p21, PUMA, and MDM2, in CRC cells (Fig. 3a, b). Moreover, we noted that alterations in wt p53 expression were able to significantly modulate miR-149-3p expression, as shown in Fig. 3c, d, indicating that miR-149-3p was positively regulated by wt p53. Therefore, using the p53-null HCT116 cell line (TP53^−/−^), we found that miR-149-3p was not upregulated by DCA treatment, but ectopic expression of p53 reversed this effect. Moreover, we used nutlin-3, a potent inhibitor that inhibits the MDM2-p53 interaction, leading to the activation of p53 as a positive control, along with the expression of miR-149-3p being elevated in the wt HCT116 cell line (TP53^+/+^) (Fig. 3e–g). Mechanistically, four putative p53 binding sites to the miR-149 flanking genomic DNA region were predicted using bioinformatics analysis software (IGV). ChIP assays were then performed in the cells using an antibody against wt p53. The pulled-down DNA was amplified by ordinary PCR with primers that were designed based on these sites. Our results showed that compared with region4, the region 3 was markedly enriched after DCA treatment in wt p53-immunoprecipitated HCT116 chromatin (Fig. 3h), suggesting only region 3 contains a specific binding site activated by DCA. These results indicate that DCA modulates miR-149-3p through wt p53.

### PDK2 is a direct target of miR-149-3p

**Fig. 4:**
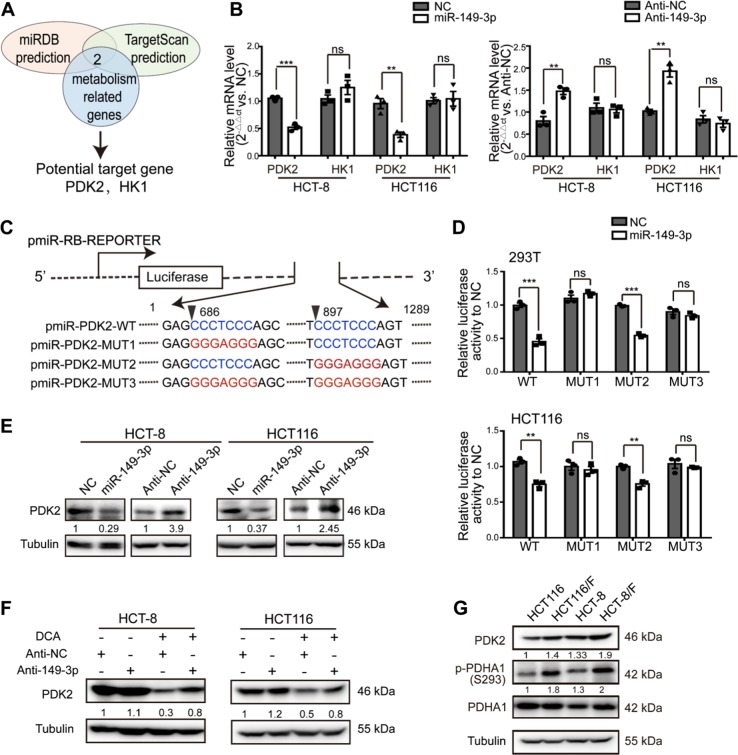
PDK2 is a direct target of miR-149-3p. **a** Schematic diagram of the protocol used to search for candidate target genes which are predictively regulated by miR-149-3p. **b** HCT-8 and HCT116 cells were transiently transfected with an miR-149-3p mimic or inhibitor. The mRNA levels of PDK2 and HK1 were analyzed by quantitative real-time PCR. **c** Diagram of the miR-149-3p putative binding sites in the 3′-UTR of PDK2. The mutant sequences used in the luciferase reporters are indicated in red. **d** Human embryonic kidney 293 T cells and HCT116 cells were cotransfected with luciferase reporter constructs and the miR-149-3p mimic (50 nmol/L) or miRNA control for 48 h. Relative luciferase activity data were normalized to corresponding control. **e** HCT-8 and HCT116 cells were transfected with either the miR-149-3p mimic or inhibitor for 48 h, and the expression of PDK2 was determined by Western blot analysis. **f** HCT116 cells transfected with the miR-149-3p inhibitor or anti-NC were treated with or without DCA for 24 h. The expression of PDK2 was determined by Western blot analysis. **g** The expression of PDK2 and p-PDHA1 in chemosensitive and chemoresistant CRC cells were analyzed by Western analysis. Data shown are representative pictures of three experiments. The results of three independent experiments performed in triplicate are shown as the mean ± SEM. ^*^P *<* 0.05; ^**^P *<* 0.01; ^***^P *<* 0.001

To elucidate the mechanisms by which miR-149-3p regulates chemosensitivity in CRC cells, we assayed genes associated with energy metabolism, which are regulated by miR-149-3p using two public platforms (TargetScan and miRDB). Finally, we identified that pyruvate dehydrogenase kinase 2 (PDK2) and hexokinase 1 (HK1) are potential candidates (Fig. 4a). To confirm these findings, the miR-149-3p mimic or inhibitor was transfected into CRC cells. We found that the mRNA levels of PDK2 were negatively regulated by miR-149-3p, but not by HK1 (Fig. 4b and Supplementary Fig. 3A). Two possible miR-149-3p binding sites in the 3′-UTR of PDK2 were found, and the dual-luciferase reporter assay indicated that miR-149-3p binds to the predicted site (686-693) of the PDK2 3′-UTR (Fig. 4c, d). We then confirmed that the PDK2 protein levels were negatively regulated by miR-149-3p (Fig. 4e).

PDK has four isozymes named PDK1, 2, 3, and 4, all of which have been reported to be regulated by DCA [26, 38, 39]. We then analyzed the mRNA expression of PDK1–4 in both HCT8 and HCT116 cells after DCA treatment. DCA significantly inhibited the mRNA expression of PDK2, but not other PDK isozymes (Supplementary Fig. S3B). Furthermore, the transfection of anti-149-3p partially reversed the reduction of PDK2 by DCA (Fig. 4f). Next, we determined PDK2 and its downstream pyruvate dehydrogenase E1-alpha subunit (PDHA1) protein levels in both 5-FU-sensitive and 5-FU-resistant CRC cells. The basal levels of PDK2 were elevated in chemoresistant CRC cells, compared with the levels in chemosensitive cells. In line with this elevation, the phosphorylation of PDHA1 was elevated as well (Fig. 4g).

### The miR-149-3p/PDK2 pathway regulates chemosensitivity

**Fig. 5:**
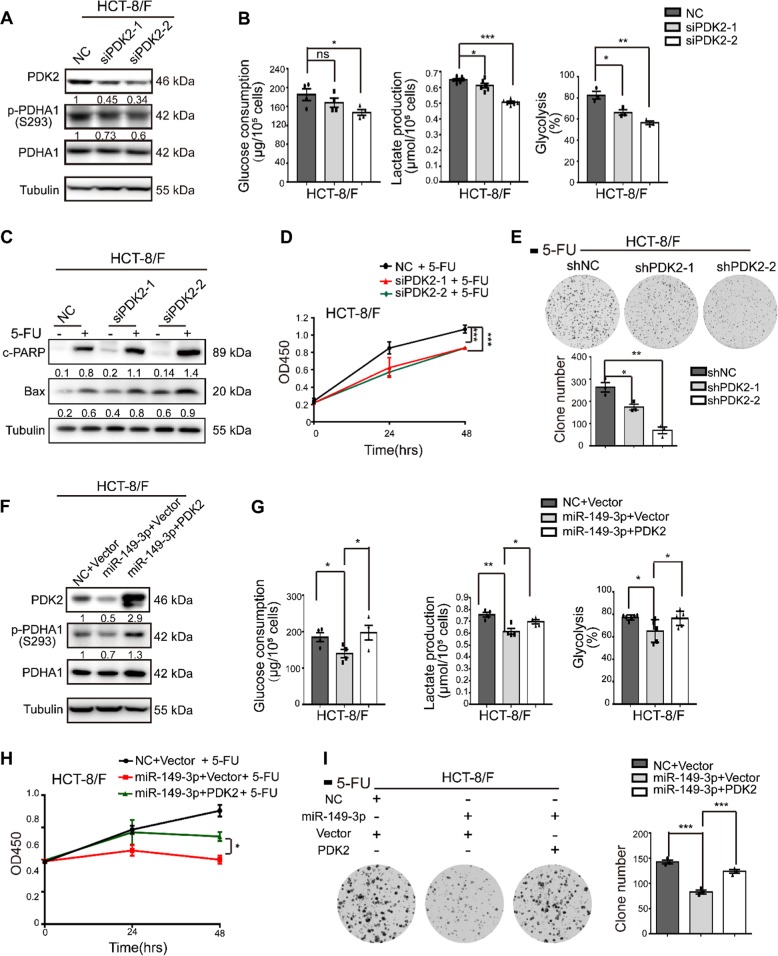
miR-149-3p/PDK2 signaling inhibits glucose metabolism and increases chemosensitivity. **a** PDK2 was knocked down by transient transfection with an siRNA in HCT-8/F cells. The knockdown resulted in the alteration of p-PDHA1. **b** The glucose consumption, lactate production and glycolysis of CRC cells transfected with PDK2 siRNA or NC were measured. **c** HCT-8/F cells were treated with or without 50 μg/ml 5-FU for 24 h after transfection with PDK2 siRNA or NC. The recognized biomarkers (c-PARP, Bax) for cell apoptosis were determined by Western blot analysis. **d** HCT-8/F cells transfected with PDK2 siRNA or NC were treated with 5-FU. The cell growth was determined by a CCK8 assay. **e** HCT-8/F cells infected with PDK2 shRNA or the negative control virus were treated with 5-FU. The colony formation capacity was determined by crystal violet staining. **f** HCT-8/F cells were transfected with NC or the miR-149-3p mimic for 24 h and then infected with a virus overexpressing PDK2. The expression of PDK2 and p-PDHA1 was determined by Western blot analysis. **g** Glucose consumption, lactate production and glycolysis were measured. **h**, **i** After infection, cells were treated with 5-FU and the cell growth and capacities of colony formation were measured. Representative results of three independent experiments performed in at least triplicate are shown as the mean ± SEM. ^*^P *<* 0.05; ^**^P *<* 0.01; ^***^P *<* 0.001

To understand how the miR-149-3p/PDK2 pathway regulates the CRC cell response to 5-FU, the HCT-8/F cell line was selected as a representative cell line to investigate whether the expression levels of miR-149-3p and PDK2 affected the cell response to 5-FU. Knockdown of PDK2 in HCT-8/F cells inhibited the phosphorylation of PDHA1 (Fig. 5a) and reduced energy metabolism markers, such as glucose consumption, lactate production, and glycolysis (Fig. 5b). Moreover, reductions in PDK2 were able to enhance 5-FU effects on increasing the levels of cleaved PARP (c-PARP) and Bax, both of which are recognized biomarkers for cell apoptosis in HCT-8/F cells (Fig. 5c). In addition, PDK2 knockdown increased chemosensitivity to 5-FU in HCT-8/F cells as determined by CCK8 and colony formation assays (Fig. 5d, e). Knockdown of PDK2 promoted 5-FU-induced apoptosis in HCT-8/F and HCT116/F cells and is shown in Supplementary Fig. S4A, whereas overexpression of PDK2 promoted the phosphorylation of PDHA1 and mitigated the cell apoptosis induced by 5-FU in HCT-8 and HCT116 cells (Supplementary Fig. S4B–S4C).

In addition, overexpression of PDK2 reversed the inhibitory effect of miR-149-3p on PDK2 (Fig. 5f) and partially abolished the inhibitory effect of miR-149-3p on glucose consumption, lactate production, and glycolysis (Fig. 5g. Ectopic expression of PDK2 markedly abolished the inhibitory effect of miR-149-3p on cell growth and colony formation in HCT-8/F cells treated with 5-FU (Fig. 5h, i). Taken together, our results indicate that the miR-149-3p/PDK2 pathway restores chemosensitivity by, at least partially, targeting glucose metabolism in chemoresistant CRC cells.

### DCA enhances chemosensitivity of 5-FU in vivo

**Fig. 6:**
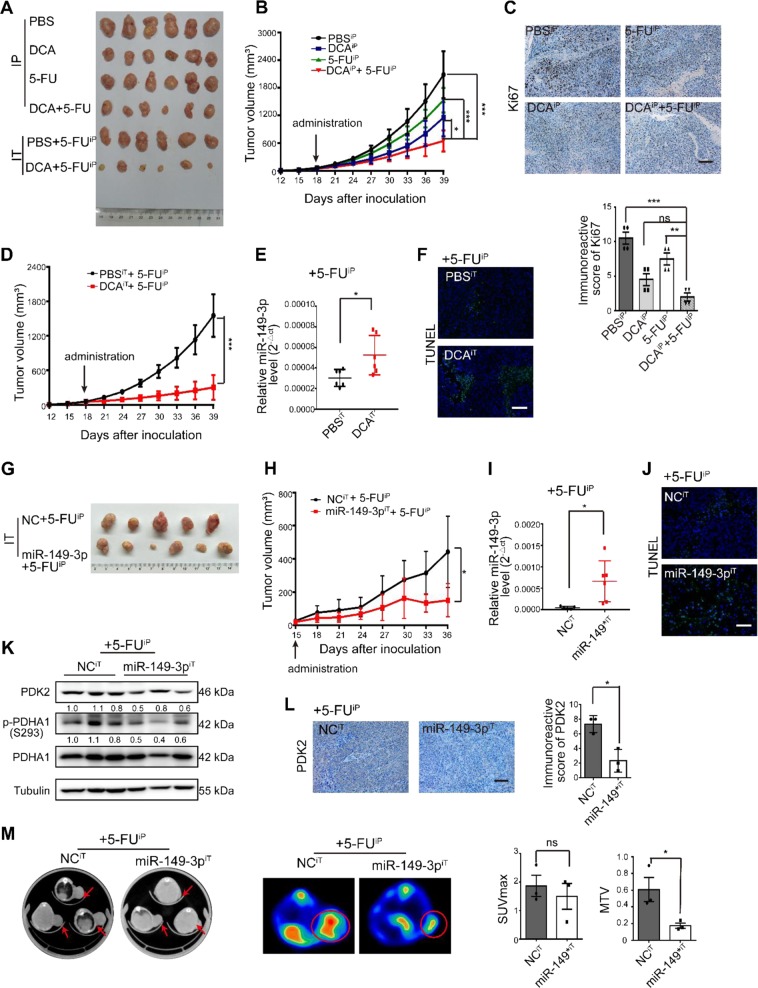
Combination treatment of 5-FU with DCA or miR-149-3p enhances chemotherapy effects in vivo. **a** Representative images of tumors in each group. **b** Xenograft tumor volumes were calculated in the intraperitoneal group. The mean ± SD are shown, *n* × 6. **c** Immunostainings of xenograft tumor sections with Ki-67 in the intraperitoneal group. Positive staining is in brown (Scale bars: 100 µm) (upper panel). The immunoreactive score of Ki67 was calculated (lower panel). The mean ± SD are shown, *n* × 4. **d** Xenograft tumor volumes were calculated up to 39 days post-tumor inoculation in the intratumoral group. **e** The expression of miR-149-3p was determined by quantitative real-time PCR in xenograft tumors after DCA intratumoral injection. The mean ± SD are shown, *n* × 6. **f** Representative TUNEL stained sections from the mice in the intratumoral group are shown (scale bars: 50 µm). **g** Representative image of tumors from mice that received an intratumoral injection of agomiR-NC or agomiR-149-3p for 3 weeks. **h** Xenograft tumor volumes were calculated up to 21 days post treatment. The mean ± SD are shown, *n* × 6. **i** The levels of miR-149-3p were analyzed by quantitative real-time PCR. The mean ± SD are shown, *n* × 5. ^*^P < 0.05. **j** Apoptosis of tumor cells was determined by the TUNEL assay (Scale bars: 50 µm). **k** Xenograft tumors from three mice in each group were analyzed by immunoblot with the indicated antibodies. **l** Images to visualize the positive staining of PDK2 in xenograft tumors (Scale bars: 100 µm) (left panel). The immunoreactive score of PDK2 was calculated (right panel). The mean ± SD are shown, *n* × 3. **m** Representative 18F-FDG micro-PET/CT images of tumor-bearing mice. Tumors are indicated by red arrows and circles. The values of SUVmax and MTV were obtained in tumors. The mean ± SD are shown, *n* × 3. ^*^P *<* 0.05; ns no significance

Next, 5-FU, DCA or a combination of 5-FU with DCA were intraperitoneally injected into the subcutaneous xenograft model (noted as the intraperitoneal group). Considering the insufficient blood supply in the central part of subcutaneous tumors, we also intratumorally injected DCA or PBS plus an intraperitoneal injection of 5-FU (noted as the intratumoral group). The combinations of DCA with 5-FU were showed better inhibitory effects on tumor growth than did DCA or 5-FU alone after four weeks of injection in the intraperitoneal group (Fig. 6a, b). The expression of Ki-67 was reduced after combination treatment in the intraperitoneal group (Fig. 6c). DCA significantly inhibited tumor growth in the intratumoral group compared with that in the PBS group (Fig. 6d), and miR-149-3p was upregulated in the DCA intratumoral injection group, which is consistent with the results in vitro (Fig. 6e). Intratumoral injection of DCA also promoted tumor apoptosis (Fig. 6F).

To further evaluate the effect of miR-149-3p on the CRC cell response to 5-FU in vivo, the subcutaneous xenograft model was subjected to intratumoral injection of agomiR-149-3p or agomiR-NC plus an intraperitoneal injection of 5-FU. miR-149-3p significantly inhibited tumor growth in the intratumoral group compared with that in the control group in which agomiR-negative control (NC) was intratumorally injected (Fig. 6g, h). Overexpression of miR-149-3p was validated by quantitative real-time PCR (Fig. 6i), and overexpression of miR-149-3p promoted apoptosis (Fig. 6j) and reduced the expression of PDK2 and p-PDHA1 (Fig. 6k, l). Using 18F-fluorodeoxyglucose (FDG) micro-positron emission tomography (PET)-CT scanning, tumor imaging was performed after 3 weeks of treatment. 18F-FDG uptake was observed at the sites of tumor implantation, and the maximum standardized uptake (SUV_max_) and metabolic tumor volumes (MTVs) were measured. No difference in SUV_max_ was observed between the two groups, while MTV was significantly decreased in the miR-149-3p group (Fig. 6m).

### miR-149-3p is inversely correlated with PDK2 in CRC patients

**Fig. 7:**
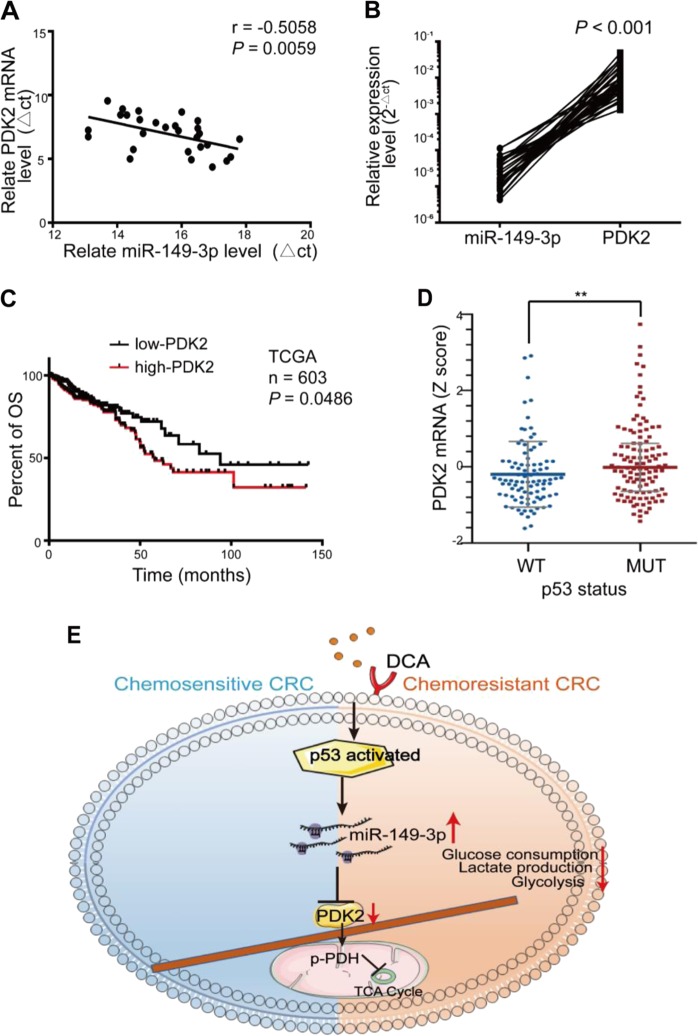
The expression of miR-149-3p is inversely correlated with PDK2 in CRC patients. **a** The correlation between the expression of miR-149-3p and PDK2 was determined using linear regression analysis (*n* × 28, *r* × −0.5058, *P* *<* 0.01) and **b** a paired *t*-test with the same samples (*P* *<* 0.001) in CRC patients. **c** The OS of CRC patients was stratified by PDK2 expression in TCGA datasets (*n* × 603, *P* × 0.0486, *P* values were obtained using the log-rank test). **d** The mRNA level of PDK2 in the wt p53 and mutant p53 groups was determined using unpaired *t*-test analysis (*P* × 0.0057) in TCGA datasets. **e** A cartoon sketch depicting DCA restores the CRC response to chemotherapy through the p53/miR-149-3p/PDK2-mediated glucose metabolism pathway

A significant inverse correlation between the miR-149-3p and PDK2 mRNA levels was observed in human CRC tissue (Fig. 7a, b). Among them, eight patients in stable condition/disease (SD) within 3 years after chemotherapy expressed a higher level of miR-149-3p than five patients with progressive disease (PD) (Supplementary Fig. S5A). Five pairs of PD and SD patients with the same pathology and TNM stage were analyzed, and PDK2 staining varied significantly in samples from the PD and SD patients (Supplementary Fig. S5B). CRC patients from TCGA database with high PDK2 expression were also characterized by a worse overall survival (OS) (Fig. 7c). The TCGA database also showed that the expression of PDK2 in the wt p53 group was reduced compared with that in the mutant p53 group (Fig. 7d). These results suggested that PDK2 is negatively regulated by miR-149-3p in CRC patients.

## Discussion

CRC is characterized by tumorigenic abnormalities and altered metabolic pathways and is one of the leading causes of death from cancers [2]. Resistance to chemotherapy is the main cause for treatment failure [7]. In this study, we found that DCA could increase the chemotherapeutic effect of 5-FU in chemoresistant CRC cells and that activation of the p53/miR-149-3p/PDK2 pathway was able to increase chemosensitivity in vitro and in vivo.

Increasing evidence indicates that increased glycolysis is closely related to resistance to chemotherapy [15, 17, 40]. Here, we also found that compared with their parental cell lines, chemoresistant CRC cells showed elevated glucose consumption, lactate production, and glycolysis, suggesting that metabolic abnormalities are a typical feature, but the molecular mechanisms still remain unclear in CRC cells.

DCA has been observed to decrease blood lactate levels in vivo in rodents at dosages of ~25–50 mg/kg/24 h [41] and to employ DCA with a wide dosage range from 1 to 50 mM [26]. Notably, the adverse effect of DCA in humans is generally limited to reversible sensory and motor peripheral neuropathy, which is influenced by age and genotype [42]. Recently, DCA has been identified as a novel metabolic therapy for various cancer patients [26, 29].

It has been reported that DCA is able to inhibit PDK activity and convert pyruvate to acetyl-CoA, leading to a shift in energy generation from glycolysis to mitochondrial oxidative phosphorylation [43, 44]. DCA has also been shown to attenuate hypoxia-induced resistance to 5-FU in gastric cancer [45], to overcome sorafenib resistance in hepatocellular carcinoma [46], and to attenuate cisplatin resistance in head and neck cancer [47]. Our study revealed that DCA was able to alleviate the chemoresistance of CRC cells to 5-FU. Moreover, we demonstrated that DCA reduced glucose consumption and lactate production in chemoresistant CRC cells to the basal level of chemosensitive CRC cells. Given that autophagy has the potential to fuel cancer metabolism [48], the effect of DCA and miR-149-3p on autophagy was determined. We found that DCA activates autophagy and miR-149-3p has no influence on autophagy. It suggested that autophagy was not involved in the effect of DCA/miR-149-3p in regulating glucose metabolism (Supplementary Figure S6).

PDKs, as the key regulators in the glycolysis of cancer, have caused great concern due to the results of many studies [49]. There are four isoforms of PDK (PDK1-4), and each of them occurs in a tissue-specific manner as follows: PDK1 is highly expressed in heart, PDK2 is ubiquitously expressed, PDK3 has a relatively limited tissue distribution, and PDK4 is expressed in the heart and skeletal muscle [27]. PDK2 is expressed at higher levels compared with other isoenzymes, suggesting that it may be the major isoform responsible for the regulation of pyruvate dehydrogenase complex (PDHC) enzymatic activity [50]. In addition, PDK isoenzymes differ in their acute regulation by metabolites [51]. Here, we focused on PDK2, the most sensitive isoforms to DCA [31]. Although the molecular interactions between DCA and PDKs have been focused on, the potential mechanism of the transcriptional regulation of PDK remains unclear. A recent study reported that miR-182 plays regulatory roles in lung cancer metabolic pathways by targeting PDK4 [52]. The present study demonstrated that PDK2 was regulated by miR-149-3p in CRC and that the levels of PDK2 in the primary tumors of CRC patients were inversely correlated with miR-149-3p expression. Since PDK2 is broadly present in most tissues, targeting PDK2 may be a more important and efficient way to kill tumor cells and overcome chemoresistance.

It has been reported that miR-149-3p plays a vital role in various cancers and is induced by some antitumor drugs [36, 37, 53]. Notably, we found that DCA treatment could induce the binding of p53 to the upstream region (−677 to −477) of miR-149 and that miR-149-3p was upregulated by DCA treatment in a p53-dependent manner. TP53, a classical tumor suppressor, is frequently inactivated in tumors [54] and has been recently reported to regulate glucose metabolism in cancer. Wt p53 was shown to be able to inhibit the “Warburg effect” by controlling PDK2. However, the frequency of TP53 mutations in CRC is ~40–50% [55, 56], resulting in loss of its suppressive function. It was reported that CRC patients with wt TP53 gain a survival benefit from 5-FU-based chemotherapy, but those with mutant TP53 do not [57]. Our results reveal a novel mechanism between p53 and PDK2 that is modulated by miR-149-3p. These findings suggest that patients with mutant TP53 may benefit more from adjunction chemotherapy with miR-149-3p rather than with DCA. Considering the high frequency of TP53 mutations in CRC, we believe that miR-149-3p plays a vital role in monitoring and modulating chemosensitivity in CRC.

Cancer cells consume a large amount of glucose and display high aerobic glycolysis state, therefore reducing glucose uptake is a promising strategy to restrict cancer growth [58]. We observed that the elevation of miR-149-3p remarkably inhibited glycolysis in chemoresistant CRC cells; however, compared with the control group, the xenograft group had the miR-149-3p mimic injected intratumorally and had a SUVmax that remained unchanged. Perhaps this contradictory result might be due to the development of necrosis in the core part of subcutaneous tumor tissues, which warrants investigation.

Taken together, we disclose that the p53/miR-149-3p/PDK2 signaling pathway can potentially be targeted in overcoming chemoresistant CRC upon DCA treatment, providing a potential strategy for CRC treatment from the angle of intervening in tumor metabolism (Fig. 7e).

## Materials and methods

### Cancer tissue

Twenty-eight CRC patients from the Ninth People's Hospital Affiliated to Shanghai Jiao Tong University School of Medicine were included between 2013 and 2016. Thirteen of these patients received 5-FU-based postoperative chemotherapy and were followed up for at least 3 years. All tissues were collected after getting informed consent, and all procedures involving human patients were conducted in accordance with the regulations set forth by the Ethical Committee of the Ninth People's Hospital Affiliated to the Medical College of Shanghai Jiao Tong University. The clinical information of the CRC patients is presented in Supplementary Table 1.

### Cell culture

The 5-FU-resistant cell line HCT-8/F and its parental HCT-8 cell line were purchased from iCell Bioscience, Inc. (Shanghai, China). The 5-FU-resistant cell line HCT116/F and its parental HCT116 cell line were kindly provided by Dr Gu (Yanhong Gu, Nanjing Medical University, Jiangsu, China). HCT116^−/−^ cells were a gift from Dr. Lu (Hua Lu, Fudan University, Shanghai, China). The human embryonic kidney 293T cell line was obtained from American Type Culture Collection (ATCC, Manassas, VA, USA). The 293T, HCT116, HCT-8 cell lines were cultured in Dulbecco’s Modified Eagle’s medium (HyClone, Utah, US) or RPMI-1640 medium (HyClone, Utah, US) containing 10% fetal bovine serum (Gemini, California, US), 100 U/mL penicillin, and 100 μg/mL streptomycin (HyClone, Utah, US) in a 37 °C, 5% CO_2_ humidified incubator. The culture media of the HCT-8/F and HCT116/F cell lines were supplemented with 15 μg/ml 5-FU and 5 μg/ml 5-FU, respectively. All cell lines were authenticated by short tandem repeats sequencing by Genetic Testing Biotechnology Corporation (Suzhou, Jiangsu, China). DCA was purchased from Sigma-Aldrich Co. Ltd. (MO, USA).

### Immunofluorescence staining of Edu and ROS

HCT-8/F and HCT116/F cells were seeded into 96-well plates at 15,000 cells/well. After overnight incubation, the cells were treated with 15 mM and 20 mM DCA, respectively, for 24 h. Edu staining was performed according to the manufacturers’ instructions (Ribobio, Guangzhou, China). The levels of ROS were measured in cells incubated with 10 μM 2′,7′-dichlorofluorescein diacetate (DCF-DA) (Beyotime, Shanghai, China) for 30 min at 37 °C. The plates were then washed twice and the cells were analyzed using a fluorescence microscope.

### Cell growth

Cells were seeded into 96-well plates at 5000 cells/well for overnight, treated with drugs for 24 h, and each well was subsequently replaced with mixtures of 10 μl of CCK8 (Dojindo, Japan) and 90 μl of culture medium. Absorbance was measured at an OD value of 450 nm using an enzyme microplate reader (BioTeck, Vermont, US) two hours later. The inhibition ratio of the drug was calculated with the following formula: 1-OD_drug_/OD_ctrl._ The IC50 of each cell was calculated by GraphPad Prism 6 (GraphPad Software, San Diego, CA).

### Cell apoptosis assay

HCT-8/F and HCT116/F cells were seeded in six-well plates at a concentration of 2 × 10^5^ cells/well. The cells were treated with DCA (15 mM) / 5-FU (50 μg/ml) and DCA (20 mM) /5-FU (25 μg/ml), respectively, for 48 h. The cells were then trypsinized, washed, and stained with Annexin V-FITC/PI or Annexin V-PE/7-AAD antibodies according to the manufacturer’s protocol (BD, CA, USA). Apoptosis was measured by flow cytometry (BD, CA, USA).

### Colony formation assay

HCT-8/F and HCT116/F cells were treated with DCA (15 mM)/5-FU (50 μg/ml) and DCA (20 mM)/5-FU (25 μg/ml) respectively for 24 h. Then, the cells were seeded into six-well plates at 1000 cells per well and cultured in fresh medium at 37 °C for 1 to 2 weeks, followed by fixation with 4% paraformaldehyde for 30 min; cells were then stained with 1% crystal violet, and the cell colony numbers were counted by a counter (Gelcount, Optronix, Oxford).

### Transient gene transfection

miR-149-3p mimics, inhibitors, siPDK2, sip53, and their corresponding NC oligonucleotide sequences were synthesized by GenePharma (Shanghai, China). Flag-p53 plasmid was kindly provided by Dr. Lu (Hua Lu, Fudan University, Shanghai, China). Transfection was performed with Lipofectamine 3000 (Invitrogen, CA, USA) at a final concentration of 50 nmol/L (mimics and siRNAs) or 100 nmol/L (inhibitors). Cells were harvested for assays 24 or 48 h after transfection. The siRNA, mimic, and inhibitor sequences are shown in Supplementary Table 2.

### Stable gene transfection

LV-PDK2 and the corresponding NC virus were purchased from GeneChem (Shanghai, China). shPDK2-1, shPDK2-2, and the control plasmid were purchased from GeneChem (Shanghai, China). The virus supernatant was harvested from 293 T cells. Subsequently, CRC cells were infected with the virus and screened with puromycin. The efficiency of infection was validated by flow cytometry and fluorescence microscopy. The mRNA and protein expression of PDK2 were further analyzed by quantitative real-time PCR and Western blot.

### 3′-UTR reporter luciferase assays

The wt or mutant miR-149-3p binding sequences in the human PDK2 3′UTR were cloned into downstream of the luciferase pmiR-RB-Reporter (Ribobio, Guangzhou, China), refer to WT, MUT1, MUT2, and MUT3 in Fig. 3c. 293 T cells and HCT116 cells were seeded into 24-well plates followed by cotransfection with 500 ng reporter constructs and either 50 nmol/L miR-149-3p mimic or a NC using Lipofectamine 3000 (Thermo Fisher Scientific, Waltham MA). Luciferase activity was measured after 48 h of incubation using the Dual-Luciferase Reporter Assay System (Promega, Madison, USA) according to the manufacturer’s protocol.

### Glucose consumption and lactate production

HCT-8/F and HCT116/F cells were seeded into 24-well plates at 1 × 10^5^ cells/well overnight, and then treated with 15 mM and 20 mM DCA, respectively, for 24 h. After treatment, cells were cultured in phenol red-free medium containing 10% fetal bovine serum for 24 h. The cultured medium was harvested, and glucose consumption and lactate production were measured. The lactate was measured using a Lactate Assay Kit (Njjcbio, Nanjin, China), and the glucose was measured by a Glucose Assay Kit (Rsbio, Shanghai, China). All values were standardized by counting an equal number of cells. To evaluate the glycolysis state, 100 ng/mL oligomycin (an ATP synthase inhibitor; Sangon Biotech) was added to cultured cells for 6 h. The ratio of the lactate concentration in the presence and absence of oligomycin was measured and determined as described previously [46].

### Seahorse XF-96 glycolytic rate assay

Cells were seeded into a 96-well culture plate at a density of 25,000 cells/well and were incubated overnight in growth medium containing 10% fetal bovine serum. Sensor cartridge was hydrated overnight. Next day, cells medium was changed to bicarbonate-free low-buffered assay medium supplemented with glucose, sodium pyruvate, and glutamin. After cells incubated for 1 h at 37 °C in a non-CO_2_ incubator, oxygen consumption rate and extracellular acidification rate were measured before and after the injection of DCA/ctrl, Rotenone (Rot) + antimycin A (AA) and 2-deoxy-d-glucose (2-DG) using the Seahorse XF instrument (Agilent, Santa Clara, CA) as previously described [59, 60]. Experiments were performed in real time in five to six replicate wells. GlycoPERs include basal glycoPER, induced glycoPER, and compensatory glycoPER were automatically calculated by the Wave software (Agilent, Santa Clara, CA).

### Quantitative real-time PCR and miRNA microarray

Total RNA was extracted from CRC tissues or cells with TRIzol Reagent (Life, CA, USA). cDNA was synthesized using the PrimeScript RT Reagent Kit (TaKaRa, Tokyo, Japan). The microarray assay was performed with three replicates HCT116 cells treated with 5 mM, 10 mM, or 20 mM DCA for 12, 24, or 48 h. The original data were uploaded to GEO database (GSE125309). Quantitative real-time PCR was performed using premix Ex Taq 420 A (TaKaRa, Tokyo, Japan) on the ABI-7500 platform. Actin and U6 were used as internal controls. Primer sequences are presented in Supplementary Table 3.

### Western blots

Thirty micrograms of total protein lysates were loaded, and the primary antibodies were applied: anti-PDK2 (sc-100534, Santa Cruz, California, US), anti-p-PDHA1 (S293) (ABS204, Merck, Darmstadt, Germany), anti-PDHA1 (ab168379, Abcam, Cambridge, UK), anti-p53 (sc-126, Santa Cruz, California, US), anti-c-PARP (D64E10, CST, Massachusetts, US), anti-Bax (D2E11, CST, Massachusetts, US), anti-LC3B (L7543, sigma, MO, USA), anti-GAPDH (Proteintech, Wuhan, China), and anti-α-tubulin (Proteintech, Wuhan, China). The secondary antibodies were purchased from Sungene (Tianjin, China). The blot assays were imaged by a chemiluminescence imaging system (Bioshine, Shanghai, China).

### Chromatin immunoprecipitation (ChIP)

HCT116 cells seeded in 10 cm plates were treated with or without 20 mM DCA for 24 h, and then cell fixation and chromosome fragmentation were performed according to the manufacturer’s instructions (Pierce Agarose ChIP Kit, Thermo). The chromatin was incubated with IgG and anti-p53 antibodies (Sigma, MO, USA) at 4°Covernight. After incubation, 60 ul protein A agarose/salmon sperm DNA was added. Then, the precipitated complex was washed with IP wash buffers 1, 2, 3, and eluted with elution buffer. The cross-linking was reversed by adding 6 μl of 5 M NaCl and 2 ul of proteinase K at 65°C for 1.5 h. The immunoprecipitated DNA and whole-cell extract DNA (input) were purified and then used for PCR analyses using the relevant primers. A control primer was used for monitoring the experiment. The primer sequences for PCR are presented in Supplementary Table 3.

### Subcutaneous tumor xenograft in nude mice and micro-PET/CT imaging

First, 1 × 10^7^ HCT-8/F cells diluted in 100 μl of PBS were subcutaneously implanted into nude mice (male, 6 weeks). Mice were randomly divided into six groups (six per group) after 12 days. Mice from group I to group IV received a daily intraperitoneal injection of PBS, DCA (50 mg/kg)/PBS, 5-FU (10 mg/kg)/PBS, and 5-FU (10 mg/kg)/DCA (50 mg/kg), respectively. Mice from group V and group VI were intratumorally injected with PBS or DCA (50 mg/kg), respectively, every other day and an intraperitoneal injection of 5-FU (10 mg/kg) every other day. Tumor volume was measured blindly every 3 days. Mice were sacrificed after 3 weeks of treatment, and the tumors were dissected, weighed, and frozen at −80 °C for further study.

To evaluate whether miR-149-3p exerts a chemo-sensitization effect, another two groups of animal models were established. Briefly, mice were subcutaneously implanted with 6 × 10^6^ HCT-8/F cells. After the tumor sizes were ~50 mm^3^, mice received an intraperitoneal dosage of 5-FU (10 mg/kg) every other day as well as an intratumoral injection of 5 nmol cholesterol-conjugated miR-149-3p mimics or a NC every 3 days for 3 weeks. Later, three mice from each group were fasted overnight and were intravenously injected with 0.15 mCi 18F-FDG. 18F-FDG micro-PET-CT scanning (Siemens, Berlin, Germany) was performed after 60 min. PET acquisition images were shown using a pseudocolor map with red color indicating high 18F-FDG uptake. SUVmax and MTV were used to determine 18F-FDG-PET activity. All experiments and animal care were approved by the Ethical Committee of the Ninth People's Hospital Affiliated to the Medical College of Shanghai Jiao Tong University.

### Immunohistochemical and immunofluorescence staining

Briefly, tissue sections were incubated with the primary antibodies again Ki67 (Servicebio, Wuhan, China) and PDK2 (Proteintech, Wuhan, China) at 4 °C overnight, and then incubated with the secondary antibody. The chromogenic reaction was performed with 3,3-diaminobenzidine and counterstained with hematoxylin. The immunoreactive score (IRS) was calculated by two investigators blinded to the group assignment. IRS = SI (staining intensity) × PP (percentage of positive cells). SI was assigned as follows: 0 × negative; 1 × weak; 2 × moderate; 3 × strong. PP is defined as 0 × 0%; 1 × 0–25%; 2 × 25–50%; 3 × 50–75%; 4 × 75–100%. Six-millimeter frozen sections were stained using a TUNEL reaction kit (Roche, Basel, Switzerland) and counterstained with DAPI. Images were captured using a fluorescence microscope with appropriate excitation and emission filters.

### Statistical analysis

Data were analyzed by GraphPad Prism 6.0 software. Data are presented as the means ± SD/SEM from three independent experiments. Each experiment was performed at least three replicates. Two-tailed Student’s *t* test was used to compare differences between the two groups. One-way ANOVA followed by Bonferroni’s post-hoc test was used for multiple comparisons. The Kaplan–Meier curves for survival analyses were determined using the log-rank test. The relationship between miR-149-3p and PDK2 was evaluated using Spearman’s rank correlation coefficient analysis. A *P* value <0.05 was considered statistically significant.

## Supplementary information

Supplementary Figures

Supplementary table 1

Supplementary table 2

Supplementary table 3

## References

[1] Chen W, Sun K, Zheng R, Zeng H, Zhang S, Xia C (2018). Cancer incidence and mortality in China, 2014. Chin J Cancer Res.

[2] Siegel RL, Miller KD, Jemal A (2018). Cancer statistics, 2018. CA: Cancer J Clin.

[3] Allen KT, Chin-Sinex H, DeLuca T, Pomerening JR, Sherer J, Watkins JB (2015). Dichloroacetate alters Warburg metabolism, inhibits cell growth, and increases the X-ray sensitivity of human A549 and H1299 NSC lung cancer cells. Free Radic Biol Med..

[4] Benci JL, Xu B, Qiu Y, Wu TJ, Dada H, Twyman-Saint Victor C (2016). Tumor interferon signaling regulates a multigenic resistance program to immune checkpoint blockade. Cell..

[5] Morgan RA, Yang JC, Kitano M, Dudley ME, Laurencot CM, Rosenberg SA (2010). Case report of a serious adverse event following the administration of T cells transduced with a chimeric antigen receptor recognizing ERBB2. Mol Ther: J Am Soc Gene Ther.

[6] Miller KD, Siegel RL, Lin CC, Mariotto AB, Kramer JL, Rowland JH (2016). Cancer treatment and survivorship statistics, 2016. CA: Cancer J Clin.

[7] Hammond WA, Swaika A, Mody K (2016). Pharmacologic resistance in colorectal cancer: a review. Therapeutic Adv Med Oncol.

[8] Douillard JY, Cunningham D, Roth AD, Navarro M, James RD, Karasek P (2000). Irinotecan combined with fluorouracil compared with fluorouracil alone as first-line treatment for metastatic colorectal cancer: a multicentre randomised trial. Lancet..

[9] Saltz LB, Cox JV, Blanke C, Rosen LS, Fehrenbacher L, Moore MJ (2000). Irinotecan plus fluorouracil and leucovorin for metastatic colorectal cancer. Irinotecan Study Group. New Engl J Med.

[10] Hanahan D, Weinberg RA (2011). Hallmarks of cancer: the next generation. Cell..

[11] Matthew G, Vander Heiden LCC, Craig BT (2009). Understanding the Warburg effect: the metabolic requirements of cell proliferation. Science.

[12] Adekola K, Rosen ST, Shanmugam M (2012). Glucose transporters in cancer metabolism. Curr Opin Oncol.

[13] Shaw RJ (2006). Glucose metabolism and cancer. Curr Opin Cell Biol.

[14] Van Dang C, Pollak M (2013). Why cancer & metabolism?. Why now? Cancer Metab.

[15] Zhao Y, Butler EB, Tan M (2013). Targeting cellular metabolism to improve cancer therapeutics. Cell Death Dis.

[16] Cairns RA, Harris IS, Mak TW (2011). Regulation of cancer cell metabolism. Nat Rev Cancer.

[17] Xu RH, Pelicano H, Zhou Y, Carew JS, Feng L, Bhalla KN (2005). Inhibition of glycolysis in cancer cells: a novel strategy to overcome drug resistance associated with mitochondrial respiratory defect and hypoxia. Cancer Res..

[18] Guo WQZ, Wang Z (2015). MiR-199a-5p is negatively associated with malignancies and regulates glycolysis and lactate production by targeting hexokinase 2 in liver cancer. Hepatology..

[19] Qiu Z, Guo W, Wang Q, Chen Z, Huang S, Zhao F (2015). MicroRNA-124 reduces the pentose phosphate pathway and proliferation by targeting PRPS1 and RPIA mRNAs in human colorectal cancer cells. Gastroenterology..

[20] Chen D, Wang H, Chen J, Li Z, Li S, Hu Z (2018). MicroRNA-129-5p regulates glycolysis and cell proliferation by targeting the glucose transporter SLC2A3 in gastric cancer cells. Front Pharmacol.

[21] Bartel DP (2004). MicroRNAs: genomics, biogenesis, mechanism, and function. Cell..

[22] Garzon R, Calin GA, Croce CM (2009). MicroRNAs in Cancer. Annu Rev Med.

[23] Huang S, He X (2010). microRNAs: tiny RNA molecules, huge driving forces to move the cell. Protein Cell..

[24] Zhang Y, Wang J (2017). MicroRNAs are important regulators of drug resistance in colorectal cancer. Biol Chem.

[25] Stacpoole PW, Nagaraja NV, Hutson AD (2003). Efficacy of dichloroacetate as a lactate-lowering drug. J Clin Pharmacol.

[26] Kankotia S, Stacpoole PW (2014). Dichloroacetate and cancer: new home for an orphan drug?. Biochim Biophys Acta.

[27] Bowker-Kinley MM, Davis WI, Wu P, Harris RA, Popov KM (1998). Evidence for existence of tissue-specific regulation of the mammalian pyruvate dehydrogenase complex. Biochemical J.

[28] Bonnet S, Archer SL, Allalunis-Turner J, Haromy A, Beaulieu C, Thompson R (2007). A mitochondria-K + channel axis is suppressed in cancer and its normalization promotes apoptosis and inhibits cancer growth. Cancer cell.

[29] Michelakis ED, Sutendra G, Dromparis P, Webster L, Haromy A, Niven E (2010). Metabolic modulation of glioblastoma with dichloroacetate. Sci Transl Med.

[30] Chu QS, Sangha R, Spratlin J, Vos LJ, Mackey JR, McEwan AJ (2015). A phase I open-labeled, single-arm, dose-escalation, study of dichloroacetate (DCA) in patients with advanced solid tumors. Invest New Drugs.

[31] Papandreou I, Goliasova T, Denko NC (2011). Anticancer drugs that target metabolism: Is dichloroacetate the new paradigm?. Int J Cancer.

[32] Michelakis ED, Webster L, Mackey JR (2008). Dichloroacetate (DCA) as a potential metabolic-targeting therapy for cancer. Br J Cancer.

[33] Madhok BM, Yeluri S, Perry SL, Hughes TA, Jayne DG (2010). Dichloroacetate induces apoptosis and cell-cycle arrest in colorectal cancer cells. Br J Cancer.

[34] Shahrzad S, Lacombe K, Adamcic U, Minhas K, Coomber BL (2010). Sodium dichloroacetate (DCA) reduces apoptosis in colorectal tumor hypoxia. Cancer Lett.

[35] Bertoli G, Cava C, Castiglioni I (2015). MicroRNAs: new biomarkers for diagnosis, prognosis, therapy prediction and therapeutic tools for breast cancer. Theranostics.

[36] Cao D, Jia Z, You L, Wu Y, Hou Z, Suo Y (2016). 18beta-glycyrrhetinic acid suppresses gastric cancer by activation of miR-149-3p-Wnt-1 signaling. Oncotarget..

[37] Si L, Xu L, Yin L, Qi Y, Han X, Xu Y (2017). Potent effects of dioscin against pancreatic cancer via miR-149-3P-mediated inhibition of the Akt1 signalling pathway. Br J Pharm.

[38] Kato M, Li J, Chuang JL, Chuang DT (2007). Distinct structural mechanisms for inhibition of pyruvate dehydrogenase kinase isoforms by AZD7545, dichloroacetate, and radicicol. Structure..

[39] Abbot EL, McCormack JG, Reynet C, Hassall DG, Buchan KW, Yeaman SJ (2005). Diverging regulation of pyruvate dehydrogenase kinase isoform gene expression in cultured human muscle cells. FEBS J.

[40] Bhattacharya B, Low SH, Soh C, Kamal Mustapa N, Beloueche-Babari M, Koh KX (2014). Increased drug resistance is associated with reduced glucose levels and an enhanced glycolysis phenotype. Br J Pharm.

[41] Stacpoole PW (1989). The pharmacology of dichloroacetate. Metab: Clin Exp.

[42] Shroads AL, Guo X, Dixit V, Liu HP, James MO, Stacpoole PW (2008). Age-dependent kinetics and metabolism of dichloroacetate: possible relevance to toxicity. J Pharmacol Exp Therap.

[43] Velpula KK, Bhasin A, Asuthkar S, Tsung AJ (2013). Combined targeting of PDK1 and EGFR triggers regression of glioblastoma by reversing the Warburg effect. Cancer Res.

[44] Kluza J, Corazao-Rozas P, Touil Y, Jendoubi M, Maire C, Guerreschi P (2012). Inactivation of the HIF-1alpha/PDK3 signaling axis drives melanoma toward mitochondrial oxidative metabolism and potentiates the therapeutic activity of pro-oxidants. Cancer Res.

[45] Xuan Y, Hur H, Ham IH, Yun J, Lee JY, Shim W (2014). Dichloroacetate attenuates hypoxia-induced resistance to 5-fluorouracil in gastric cancer through the regulation of glucose metabolism. Exp Cell Res.

[46] Shen YC, Ou DL, Hsu C, Lin KL, Chang CY, Lin CY (2013). Activating oxidative phosphorylation by a pyruvate dehydrogenase kinase inhibitor overcomes sorafenib resistance of hepatocellular carcinoma. Br J Cancer.

[47] Roh JL, Park JY, Kim EH, Jang HJ, Kwon M (2016). Activation of mitochondrial oxidation by PDK2 inhibition reverses cisplatin resistance in head and neck cancer. Cancer Lett..

[48] Kimmelman AC, White E (2017). Autophagy and tumor metabolism. Cell Metab.

[49] Sutendra G, Dromparis P, Kinnaird A, Stenson TH, Haromy A, Parker JM (2013). Mitochondrial activation by inhibition of PDKII suppresses HIF1a signaling and angiogenesis in cancer. Oncogene.

[50] Gudi R, Bowker-Kinley MM, Kedishvili NY, Zhao Y, Popov KM (1995). Diversity of the pyruvate dehydrogenase kinase gene family in humans. J Biol Chem.

[51] Sugden Mary C., Holness Mark J. (2003). Recent advances in mechanisms regulating glucose oxidation at the level of the pyruvate dehydrogenase complex by PDKs. American Journal of Physiology-Endocrinology and Metabolism.

[52] Li G, Li M, Hu J, Lei R, Xiong H, Ji H (2017). The microRNA-182-PDK4 axis regulates lung tumorigenesis by modulating pyruvate dehydrogenase and lipogenesis. Oncogene.

[53] Bellazzo A, Di Minin G, Valentino E, Sicari D, Torre D, Marchionni L (2018). Cell-autonomous and cell non-autonomous downregulation of tumor suppressor DAB2IP by microRNA-149-3p promotes aggressiveness of cancer cells. Cell Death Differ.

[54] Vazquez A, Bond EE, Levine AJ, Bond GL (2008). The genetics of the p53 pathway, apoptosis and cancer therapy. Nat Rev Drug Discov.

[55] Gnanapradeepan K, Basu S, Barnoud T, Budina-Kolomets A, Kung CP, Murphy ME (2018). The p53 tumor suppressor in the control of metabolism and ferroptosis. Front Endocrinol.

[56] Contractor T, Harris CR (2012). p53 negatively regulates transcription of the pyruvate dehydrogenase kinase Pdk2. Cancer Res..

[57] Iacopetta B (2003). TP53 mutation in colorectal cancer. Hum Mutat..

[58] Kim JW, Dang CV (2006). Cancer's molecular sweet tooth and the Warburg effect. Cancer Res..

[59] Hulse M, Caruso LB, Madzo J, Tan Y, Johnson S, Tempera I (2018). Poly(ADP-ribose) polymerase 1 is necessary for coactivating hypoxia-inducible factor-1-dependent gene expression by Epstein-Barr virus latent membrane protein 1. PLoS Pathog.

[60] Hlouschek J, Ritter V, Wirsdorfer F, Klein D, Jendrossek V, Matschke J (2018). Targeting SLC25A10 alleviates improved antioxidant capacity and associated radioresistance of cancer cells induced by chronic-cycling hypoxia. Cancer Lett..

