# Supplementary material for: Dichloroacetate restores colorectal cancer chemosensitivity through the p53/miR-149-3p/PDK2-mediated glucose metabolic pathway
Source: Oncogene. 2019 Oct 9;39(2):469–85. doi: 10.1038/s41388-019-1035-8 (PMC6949190; doi:10.1038/s41388-019-1035-8)
Supplement: Supplementary file 1 — Supplementary Figures [file 41388_2019_1035_MOESM1_ESM.pdf]

Supplementary Figure

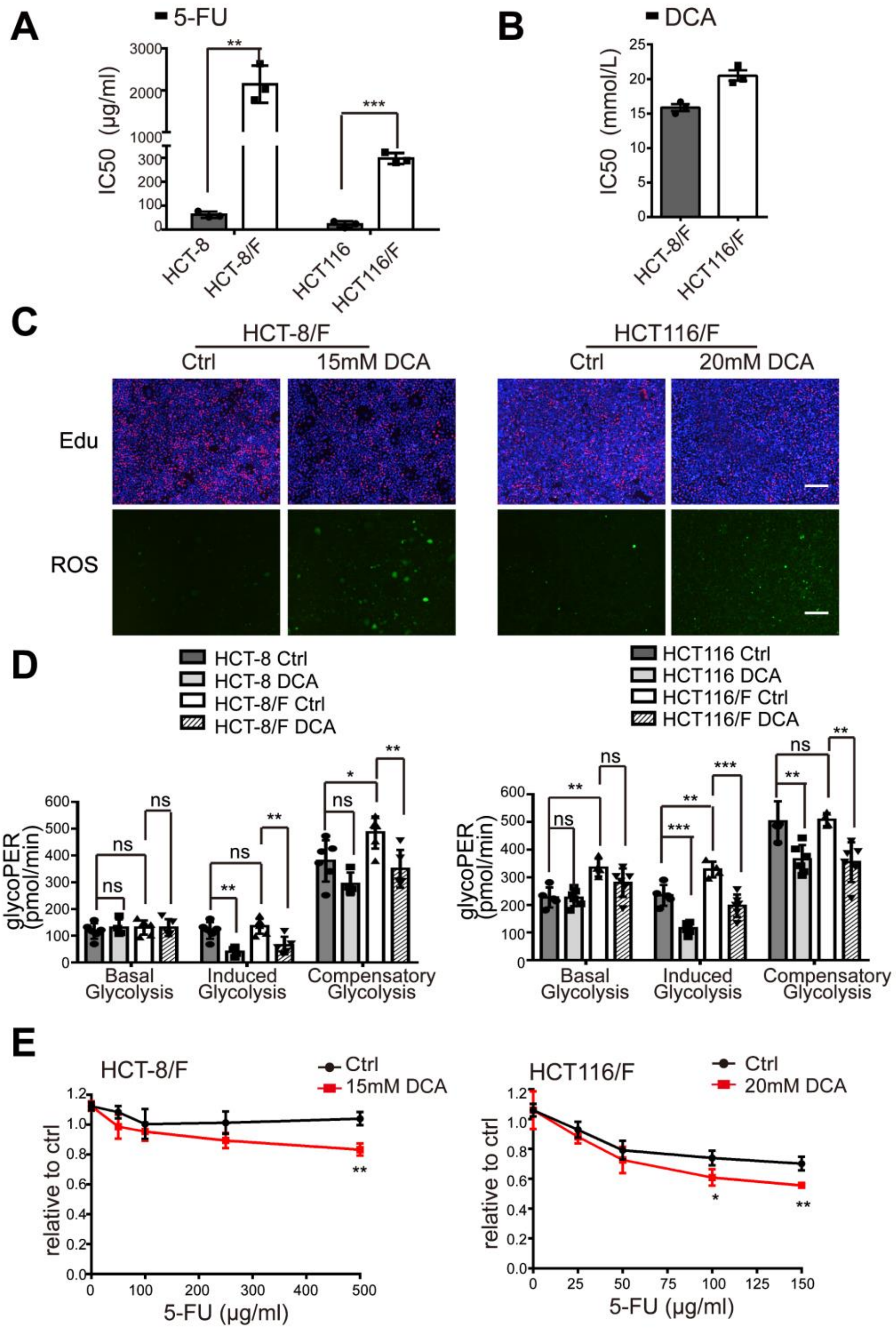

Figure S1. DCA exerts anti-tumor effect

(A) CRC cells were treated with different concentrations of 5-FU for 24 hours. The IC50 of 5-FU in each cell was calculated. (B) HCT-8/F and HCT116/F cells were treated with different concentrations of DCA for 24 hours. The IC50 of DCA in each

cell was calculated. (C) HCT-8/F and HCT116/F cells were treated with 15 mM and 20 mM DCA, respectively, for 24 hours. Representative images of Edu immunofluorescence staining (upper panel) and representative images of ROS immunofluorescence staining (lower panel), scale bar: 200 μm. (D) The determination of glycolysis rate including basal glycoPER, induced glycoPER and compensatory glycoPER were calculated by Seahorse Glycolytic Rate Assay Report Generator. (E) The cell growth was determined by a CCK8 assay after treatment with 5-FU and DCA. The dosage ranges of 5-FU were 100 ug/ml to 500 ug/ml for HCT-8/F cells and 25 ug/ml to 150 ug/ml for HCT116/F cells. Results of three independent experiments are shown as mean ± SEM. Each experiment was performed in 3-6 biological replicates. *, $P < 0.05$; **, $P < 0.01$; ***, $P < 0.001$.

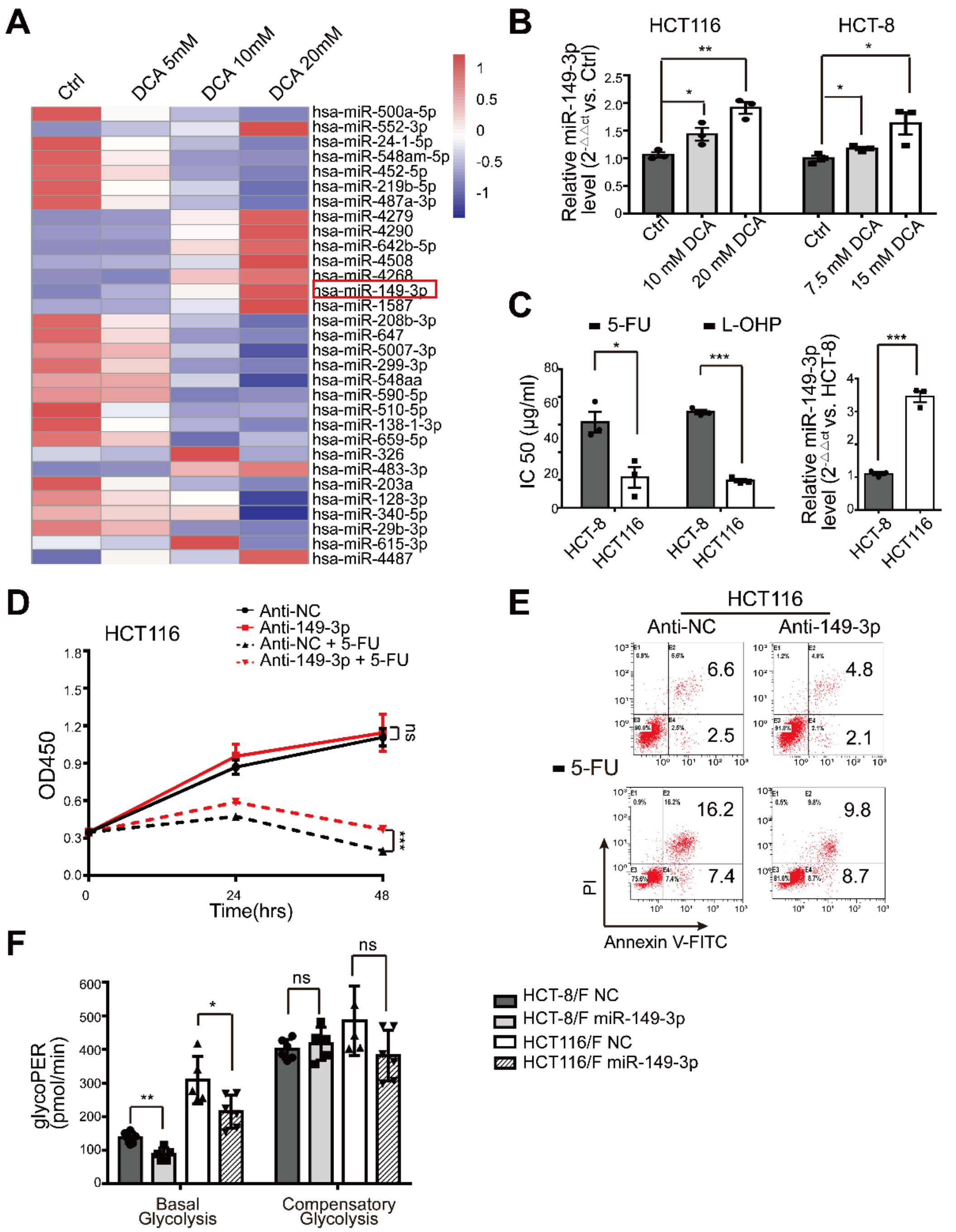

Figure S2. Knockdown of miR-149-3p decreases chemosensitivity

(A) The heatmap of differentially expressed microRNA profile in HCT116 cells treated with control, 5 mM, 10 mM, 20 mM DCA for 24 hours. (B) Total RNA was prepared at 24 hours post 10/7.5 mM and 20/15 mM DCA treatment from HCT116 and HCT-8 cells. The miR-149-3p level were analyzed by quantitative real-time PCR. (C) HCT-8 and HCT116 cells were treated with different concentrations of 5-FU and oxaliplatin

(L-OHP) respectively for 24 hours and the half maximal inhibitory concentration (IC50) was calculated (left panel). The basal levels of miR-149-3p were determined by quantitative real-time PCR in HCT-8 cells and HCT116 cells (right panel). (D-E) HCT116 cells were transiently transfected with Anti-NC or miR-149-3p inhibitor. After transfection, cells were treated with 25 μg/ml 5-FU for 24 hours. The cell growth and apoptosis were determined by CCK8 and flow cytometry respectively. (F) The determination of glycolysis rate including basal glycoPER and compensatory glycoPER in 5-FU-resistant CRC cells transfected with NC or a miR-149-3p mimic were calculated by Seahorse Glycolytic Rate Assay Report Generator. Data of three independent experiments are shown as mean ± SEM. Each experiment was performed in 3-6 biological replicates. *, $P < 0.05$; **, $P < 0.01$; ***, $P < 0.001$; ns, no significance.

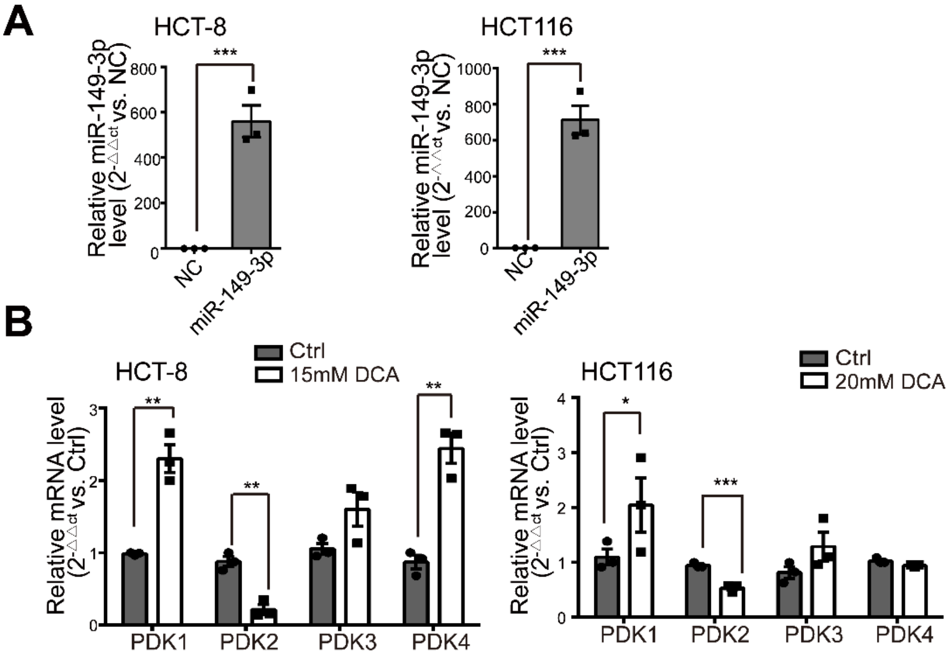

Figure S3. PDK was regulated by DCA

(A) HCT-8 and HCT116 cells were transfected with miR-149-3p mimic, and the level of miR-149-3p was measured by quantitative real-time PCR. (B) Total RNA was prepared at 24 hours post 15 mM or 20 mM DCA treatment from HCT-8 and HCT116 cells. The mRNA level of PDK1-4 were analyzed by quantitative real-time PCR. Results of three independent experiments performed in triplicate are shown as mean ± SEM. *, $P < 0.05$; **, $P < 0.01$; ***, $P < 0.001$.

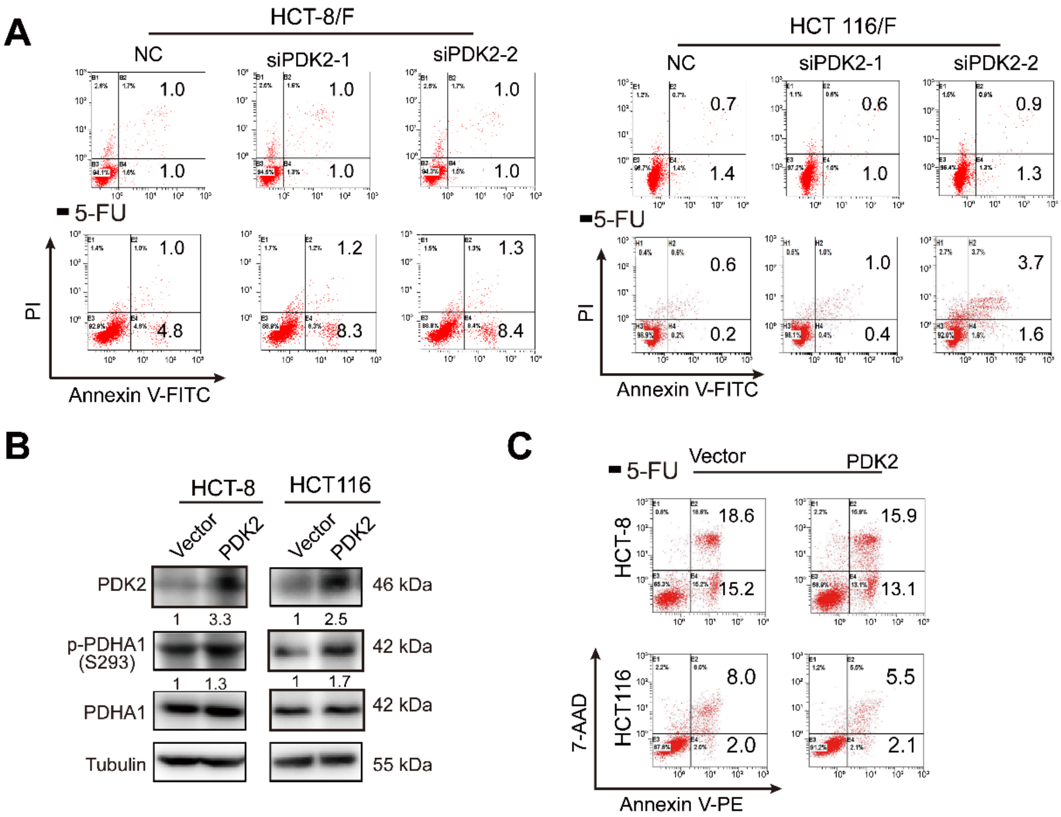

Figure S4. PDK2 is closely associated with chemosensitivity

(A) HCT-8/F and HCT116/F cells were transiently transfected with PDK2 siRNA. After transfection, cells were treated with or without 5-FU (50 µg/ml or 25 µg/ml) for 24 hours. The cell apoptosis was detected by flow cytometry. (B) HCT-8 and HCT116 cells were infected with PDK2 or control virus, and the protein level of PDK2, p-PDHA1, PDHA1 was analyzed. (C) HCT-8 and HCT116 infected with PDK2 or control virus were treated with 5-FU (50 µg/ml or 25µg/ml) for 24 hours. The apoptosis was determined by flow cytometry. Representative results of three independent experiments are shown.

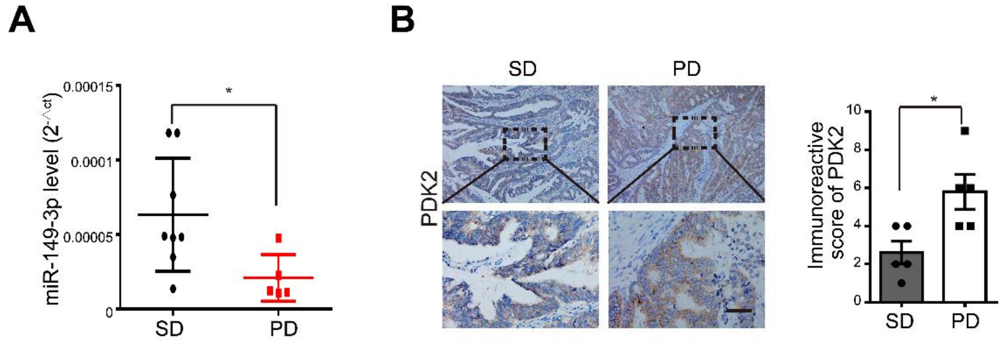

Figure S5. MiR-149-3p/PDK2 is associated with disease's progression

(A) The expression of miR-149-3p in CRC patients (n = 5 PD vs. 8 SD) who received 5-FU based chemotherapy was determined by quantitative real-time PCR. stable disease (SD), progressive disease (PD). (B) Five pairs of patients with SD and PD in same pathology and clinical stage were analyzed. Representative images of tumor tissues stained with PDK2 were shown (Scale bars: 50 μm) (left panel). The immunoreactive score of PDK2 was calculated (right panel). The mean ± SD are shown, n = 5. *, $P < .05$.

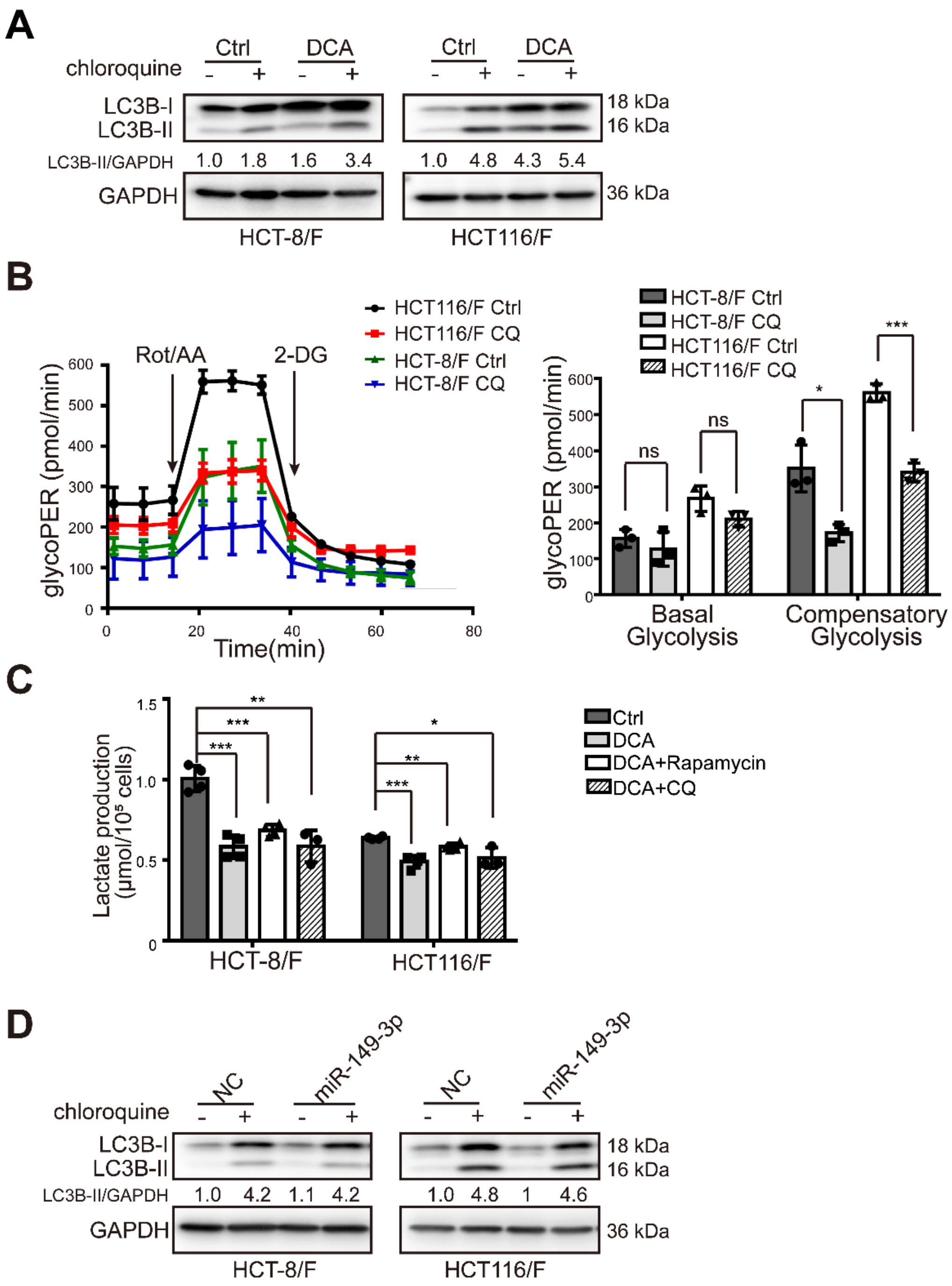

Figure S6. DCA activates autophagy and miR-149-3p has no influence on autophagy

(A) HCT-8/F cells and HCT116/F cells were treated with 20 mM and 15 mM DCA, respectively, for 24 hours with or without 50 μm chloroquine (CQ). The expression of LC3B-II was determined by Western blot analysis. (B) The determination of glycolysis rate including basal glycoPER and compensatory glycoPER in 5-FU-resistant CRC

cells treated with CQ were calculated by Seahorse Glycolytic Rate Assay Report Generator. (C) The lactate production was measured in HCT-8/F and HCT116/F cells treated with 20 mM and 15 mM DCA, respectively, in the presence and absence of rapamycin or CQ. (D) HCT-8/F and HCT116/F cells were transiently transfected with NC and a miR-149-3p mimic for 24 hours, and the cells were then treated with or without 50 μm CQ. The expression of LC3B-II was determined by Western blot analysis. Data of three independent experiments are shown as mean ± SEM. Each experiment was performed in at least three biological replicates. *, $P < 0.05$; **, $P < 0.01$; ***, $P < 0.001$; ns, no significance.
