# Supplementary material for: Dichloroacetate restores colorectal cancer chemosensitivity through the p53/miR-149-3p/PDK2-mediated glucose metabolic pathway
Source: Oncogene. 2019 Oct 9;39(2):469–85. doi: 10.1038/s41388-019-1035-8 (PMC6949190; doi:10.1038/s41388-019-1035-8)
Supplement: Supplementary file 2 — Supplementary table 1 [file 41388_2019_1035_MOESM2_ESM.doc]

**Supplementary Table 1**

| **Characteristic of CRC patients** |
| --- |
| Characteristic Total(n=28) |
| Age-yr 65.4±10.5 |
| Sex-no. (%) |
| Male 17(60.7) |
| Female 11(39.3) |
| Localization-no. (%) |
| Rectum 10(35.7) |
| Colon 18(64.3) |
| Staging- no. (%) |
| T1 or T2 6(21.4) |
| T3 or T4 22(78.6) |
| Lymph nodes involved- no. (%) |
| N0 12(42.9) |
| N1 or N2 16(57.1) |
| Metastasis- no. (%) |
| M0 27(96.4) |
| M1 1(3.6) |
| Postoperative chemotherapy with a fluorouracil-based regimen |
| Yes 21(75) |
| Follow up for 3 years |
| Yes 13(61.9) |
| No 8(38.1) |
| No 7(25) |
|  |
