# Supplementary material for: Dichloroacetate restores colorectal cancer chemosensitivity through the p53/miR-149-3p/PDK2-mediated glucose metabolic pathway
Source: Oncogene. 2019 Oct 9;39(2):469–85. doi: 10.1038/s41388-019-1035-8 (PMC6949190; doi:10.1038/s41388-019-1035-8)
Supplement: Supplementary file 3 — Supplementary table 2 [file 41388_2019_1035_MOESM3_ESM.doc]

**Supplementary Table 2:**

| **siRNA, mimic and inhibitor sequence** | |
| --- | --- |
| siPDK2-1 | sense: 5’-GACCGAUGCUGUCAUCUAUU-3’ |
|  | antisense: 5’-AAUAGAUGACAGCAUCGGUC-3’ |
| siPDK2-2 | sense: 5’-GACUCUUCAGCUACAUGUA-3’ |
|  | antisense: 5’-UACAUGUAGCUGAAGAGUC-3’ |
| NC | sense: 5’-UUCUCCGAACGUGUCACGUTT-3’ |
|  | antisense: 5’-ACGUGACACGUUCGGAGAATT-3’ |
| mimic-149-3p | sense: 5’-AGGGAGGGACGGGGGCUGUGC-3’ |
|  | antisense: 5’-ACAGCCCCCGUCCCUCCCUUU-3’ |
| inhibitor-NC | 5’-CAGUACUUUUGUGUAGUACAA-3’ |
| inhibitor-149-3p | 5’-GCACAGCCCCCGUCCCUCCCU-3’ |
| sip53 | sense: 5’-GUAAUCUACUGGGACGGAAtt-3’ |
|  | antisense: 5’-UUCCGUCCCAGUAGAUUACca-3’ |
