# Supplementary material for: Dichloroacetate restores colorectal cancer chemosensitivity through the p53/miR-149-3p/PDK2-mediated glucose metabolic pathway
Source: Oncogene. 2019 Oct 9;39(2):469–85. doi: 10.1038/s41388-019-1035-8 (PMC6949190; doi:10.1038/s41388-019-1035-8)
Supplement: Supplementary file 4 — Supplementary table 3 [file 41388_2019_1035_MOESM4_ESM.doc]

**Supplementary Table 3:**

| **primer sequences for Reverse-transcription quantitative real-time PCR** | |
| --- | --- |
| Actin | F: 5’-CTCCATCCTGGCCTCGCTGT-3’ |
|  | R: 5’-GCTGTCACCTTCACCGTTCC-3’ |
| PDK2 | F: 5’-CGCTGGCTGGCTTTGGTTATG-3’ |
|  | R: 5’-ACAGGGCCTTGAGATAGATG-3’ |
| PDK1 | F: 5’-GCTGTATGGCCTGCAAGATG-3’ |
|  | R: 5’-GCTGTCCTGGTGATTTTGCA-3’ |
| PDK3 | F: 5’-GGTTTGCCAATTTCCCGTCTG-3’ |
|  | R: 5’-CATCGGCTTCAGGCGTGGTC-3’ |
| PDK4 | F: 5’-GAGAATTATTGACCGCCTCT-3’ |
|  | R: 5’-CGAGAAATTGGCAAGCCGTAA-3’ |
| HK1 | F: 5’-CTTACTAAGGGATGCGATAAA-3’ |
|  | R: 5’-TCCCAACAATGAGTCCAACC-3’ |
| TP53 | F: 5’-CCCAAGCAATGGATGATTTGA-3’ |
|  | R: 5’-GGCATTCTGGGAGCTTCATCT-3’ |
| p21 | F: 5’-CTGGACTGTTTTCTCTCGGCTC-3’ |
|  | R: 5’-TGTATATTCAGCATTGTGGGAGGA-3’ |
| MDM2 | F: 5’-ATGAATCCCCCCCTTCCAT-3’ |
|  | R: 5’-CAGGAAGCCAATTCTCACGAA-3’ |
| PUMA | F: 5’-ACAGTACGAGCGGCGGAGACAA-3’ |
|  | R: 5’-GGCGGGTGCAGGCACCTAATT-3’ |
| miR-149-3p | RT: 5’-GTCGTATCCAGTGCAGGGTCCGAGGTATTCGCACTGG  ATACGACGCACAG-3’ |
|  | F: 5’-ACAGGGAGGGACGGGGG-3’ |
|  | R: 5’-ATCCAGTGCAGGGTCCGAGG-3’ |
| miR-128-3p | RT: 5’-TCGTATCCAGTGCAGGGTCCGAGGTATTCGCACTGGA  TACGACAAAGAG-3’ |
|  | F: 5’-CGCGTCACAGTGAACCGGT-3’ |
|  | R: 5’-AGTGCAGGGTCCGAGGTATT-3’ |
| miR-500a-5p | RT: 5’-GTCGTATCCAGTGCGTGTCGTGGAGTCGGCAATTGCAC  TGGATACGACTCTCACC-3’ |
|  | F: 5’-TAATCCTTGCTACCTGGG-3’ |
|  | R: 5’-CAGTGCGTGTCGTGGA-3’ |
| miR-552-3p | RT: 5’-GTCGTATCCAGTGCAGGGTCCGAGGTATTCGCACTGG  ATACGACTTGTCT-3’ |
|  | F: 5’-CGCGAACAGGTGACTGGTT-3’ |
|  | R: 5’-AGTGCAGGGTCCGAGGTATT-3’ |
| miR-340-5p | RT: 5’-TCGTATCCAGTGCAGGGTCCGAGGTATTCGCACTGGA  TACGACAATCAG-3’ |
|  | F: 5’-GCGCGTTATAAAGCAATGAGA-3’ |
|  | R: 5’-AGTGCAGGGTCCGAGGTATT-3’ |
| miR-29b-3p | RT: 5’-GTCGTATCCAGTGCAGGGTCCGAGGTATTCGCACTGG  ATACGACAACACT-3’ |
|  | F: 5’-CGC GCG TAG CAC CAT TTG AAA TC-3’ |
|  | R: 5’-ATCCAGTGCAGGGTCCGAGG-3’ |
| miR-326 | RT: 5’-GTCGTATCCAGTGCAGGGTCCGAGGTATTCGCACT  GGATACGACCTGGAG-3’ |
|  | F: 5’-CGC GCC TCT GGG CCC TTC-3’ |
|  | R: 5’-ATCCAGTGCAGGGTCCGAGG-3’ |
| miR-615-3p | RT: 5’-GTCGTATCCAGTGCGTGTCGTGGAGTCGGCAATTGCA  CTGGATACGACAAGAGGG-3’ |
|  | F: 5’-TCCGAGCCTGGGTCTCC-3’ |
|  | R: 5’-CAGTGCGTGTCGTGGAGT-3’ |
| U6 | F: 5’-GCTTCGGCAGCACATATACTAAAAT-3’ |
|  | RT (R): 5’-CGCTTCACGAATTTGCGTGTCAT-3’ |
| **primer sequences for PCR** | |
| Control | F: 5’-GAAGTCACTCAGCATGGAAAGAAGA-3’ |
|  | R: 5’-TCTGACAAACGTCCTTCCCCTTTCC-3’ |
| Region1 | F: 5’-ACAACGCAGGTCGCCGGGCCGGCTG-3’ |
|  | R: 5’-CATGGAGAGGTGAGGCCCGAAACAC-3’ |
| Region2 | F: 5’-GTGGGTCCGCCGGGACCTCGCGAGG-3’ |
|  | R: 5’-TCAGCTGAGATGGGCTCAGCTGTGA-3’ |
| Region3 | F: 5’-GGCTACAGCAACTTCAGCCCAGGGA-3’ |
|  | R: 5’-AGGGAGGCATTTTCCAACCTAGGGG-3’ |
| Region4 | F: 5’-AGCAACTTCAGCCCAGGGATCAG-3’ |
|  | R: 5’-TTTCCTCTTGGTCATCAGCACCT-3’ |
| Region5 | F: 5’-GGGGAAGATGGAACGAGACAGCA-3’ |
|  | R: 5’-TCCTCAGGACTGCCGCCTCAGGG-3’ |
